# A New Text Book

**Published:** 1889-02

**Authors:** 


### A New Text-Book.

The work on Operative Dentistry by Professor Thomas Fillebrown, of the Dental Department of Harvard University, is just at hand, but too late to receive an extended notice; this we will be pleased to make so soon as opportunity is had for examination. We have no hesitation in saying in advance that it will doubtless meet the expectations of the profession, for the ability of the author is everywhere recognized as of a high order. More anon.

DR. JONATHAN HUTCHINSON lately read a paper before the Clinical Society, of London, on Persisting Aptyalism, or Dry Mouth, with a report of a case. The subject was a lady of fifty, a widow in good general health, but who has passed through much trouble. The condition of aptyalism came on without any definite cause, and not very suddenly. In the course of a few months her tongue, cheeks, lips, and palate became absolutely dry. They had remained in this condition without any alleviation for about four years. The tongue was clean, red, and much sulcated. Its dryness was such that the lady had difficulty in making herself understood in talking. The pharynx was also dry, but there was no defect of secretion in the nose, and no difficulty in the flow of tears or of moisture in the conjunctivæ. She perspired freely, and, indeed, her face and forehead were liable to perspire on any slight emotional excitement. Dr. Hutchinson gave a brief summary of the cases previously recorded, five in number, and remarked upon the very close similarity which they bore to each other. All were women past middle age, and in all, the disease was persistent. In none had there followed any other disorder of the nervous system. In Hutchinson's case there was not in three or four years any depreciation of the patient's health. Other speakers present at the meeting briefly referred to the fact that they had seen five other cases in their practice. Drugs seemed powerless to affect the condition.—*Polyclinic.*

The *Popular Science News*, January, says that the body of a boy drowned at Winchendon, Mass., recently, was found through the use of the electric light, a bulb being fastened to a pole and submerged, illuminating the water for a considerable distance in the neighborhood. The electric light promises to become an important aid in all manner of submarine operations.

The *News* and the *Journal* of Chicago still continue their war against cigarettes. The *News* has had a number of cigarettes of popular brands analyzed. They were stripped of every distinguishing mark, each brand put in a separate box, the lid of which was inscribed with a letter, and the whole lot then handed over to a well-known chemist. He found that the cigarettes submitted were generally made of tobacco "imperfectly fermented," and that nearly all had an unnatural proportion of insoluble ash; that several kinds were steeped in an injurious substance, and were impregnated with dirt in varying proportions.

